# Integrated lumped parameter modeling of cardiac–vascular interaction with valve dynamics and ventricular pressure–volume

**DOI:** 10.3389/fbioe.2026.1841734

**Published:** 2026-07-13

**Authors:** Prashant Kishor Sharma, Wen Kai Yang, Chia-Yuan Chen

**Affiliations:** 1 Department of Mechanical Engineering, National Cheng Kung University, Tainan, Taiwan; 2 Department of Power Mechanical Engineering, National Tsing Hua University, Hsinchu, Taiwan

**Keywords:** aortic stenosis, aortic valve dynamics, cardiovascular system, closed-loop circulation model, hemodynamics, lumped-parameter model, time-varying elastance

## Abstract

**Introduction:**

Lumped-parameter cardiovascular models provide a useful framework for analyzing cardiac hemodynamics and valve-related flow disturbances. In this study, a lumped-parameter model was developed to investigate the hemodynamic response of the left ventricle and its interaction with aortic valve dynamics under physiological and pathological conditions.

**Methods:**

The model incorporated time-varying ventricular elastance, valve flow relations, and vascular resistance–compliance effects to simulate pressure, flow, and volume variations over a cardiac cycle. The predicted waveforms were compared with a reference model to evaluate arrangement in magnitude, waveform shape, and phase synchronization. A systematic sensitivity analysis was also performed to assess the effects of arterial compliance (C_sat_), minimum elastance (E_min_), and stenosis severity (θ_max_) on stroke volume (SV) and peak transvalvular pressure gradient (ΔP_peak_).

**Results:**

The predicted pressure, flow, and volume waveforms showed good agreement with the reference model in terms of magnitude, waveform pattern, and timing. Arterial compliance had only a minor effect on both SV and ΔP_peak_, mainly acting as a buffering parameter. Minimum elastance exerted a moderate influence on ventricular filling and pressure generation. In contrast, stenosis severity had the dominant effect on ΔP_peak_, while its influence on SV was limited. This response reflected the nonlinear pressure–flow relationship across a restricted aortic valve.

**Discussion:**

The findings demonstrated that vascular compliance, myocardial elastance, and valve geometry contributed differently to cardiovascular dynamics. The model also showed that lumped-parameter approaches can be used to study ventricular–valvular interaction and may support diagnostic analysis and patient-specific cardiovascular assessment.

## 1 Introduction

The cardiovascular system is a tightly coupled dynamical network in which cardiac contraction, vascular properties, and valve mechanics jointly govern blood flow and pressure (Menon et al., 2024; Feng et al., 2024). The left ventricle acts as the primary driving unit, while the arterial system regulates pressure through compliance and resistance. These interactions produce characteristic time-resolved waveforms of pressure, flow, and volume over each cardiac cycle, which are widely used to assess cardiovascular function (Singh et al., 2023; Kim et al., 2013; Okhovatian et al., 2022; Loganathan et al., 2024; Subendran et al., 2021). However, these variables do not evolve independently; they are intrinsically linked through ventricular elastance, valve operation, and vascular loading conditions. As a result, any alteration in one component propagates throughout the system in a coordinated manner. This interdependence motivates the development of modeling approaches capable of capturing the integrated behavior of the cardiovascular system (Blanco and Müller, 2025; Zhang et al., 2016).

To address this complexity, reduced-order modeling approaches have been developed to efficiently represent global hemodynamic behavior. Among these, zero-dimensional (0D) lumped-parameter models have been widely adopted, as they approximate the cardiovascular system using resistance, compliance, and inertance elements (Bjørdalsbakke et al., 2023; Chalumuri et al., 2024). In this framework, spatially averaged hemodynamic variables are represented at the compartment level without explicitly resolving detailed spatial flow distributions. This representation enables efficient simulation of pressure, flow, and volume relationships while preserving the essential ventricular–vascular interactions governing cardiovascular dynamics (Antón et al., 2015; Corti et al., 2011; Lu et al., 2014). Although several previous studies investigated pulse-wave propagation using one-dimensional (1D) arterial network models, the present study focused on a reduced-order 0D framework intended for system-level cardiovascular analysis with reduced computational cost. Within this framework, the time-varying elastance concept has been introduced to describe ventricular mechanics by linking chamber pressure to instantaneous volume (Munneke et al., 2022; Sharifi et al., 2024). This formulation allows reconstruction of physiologically meaningful pressure–volume relationships, including the ventricular pressure–volume loop, thereby establishing a direct relationship between measurable hemodynamic variables and underlying cardiac function (Weil et al., 2019; Scarsoglio et al., 2014; Korakianitis and Shi, 2006; Lu et al., 2016; Wang et al., 2020; Bordones et al., 2018).

Valve dynamics play a critical role in governing transitions between phases of the cardiac cycle (Tanaka et al., 2008). Valve opening and closure are driven by instantaneous pressure differences, which determine the onset of ventricular ejection and filling (Schoen, 2008; Mynard et al., 2012). This behavior directly influences transvalvular flow rates and pressure gradients, which serve as key indicators of ventricular–arterial coupling (Ding et al., 2024). These dynamics are reflected in observable features such as peak systolic pressure, pulsatile flow waveforms, and the dicrotic notch associated with valve closure (Antonini-Canterin et al., 2013). In addition, the synchronization between pressure, flow, and volume provides a comprehensive description of cardiac function. Therefore, accurate representation of valve behavior is essential for reproducing realistic cardiovascular dynamics (Laha et al., 2024).

These coupled interactions become more pronounced under pathological conditions, where structural and functional alterations modify system behavior. In particular, aortic valve stenosis introduces a geometric restriction to flow, resulting in a nonlinear increase in transvalvular pressure gradient (Franke et al., 2020). Despite this increase, stroke volume may remain relatively preserved due to compensatory mechanisms within the cardiovascular system (Stembridge and Levine, 2019). Similarly, age-related changes such as increased ventricular stiffness affect diastolic filling and pressure development, while reduced arterial compliance alters pressure buffering and waveform attenuation. These effects are clearly reflected in time-resolved hemodynamic waveforms and in the morphology of the pressure–volume loop (Danis et al., 2019; Menon et al., 2013). Therefore, the combined analysis of these variables provides critical insight into cardiovascular performance under both normal and altered physiological conditions.

Despite these advances, several limitations remain in existing lumped-parameter cardiovascular models. In many formulations, valve behavior is simplified using idealized assumptions that do not fully capture transient pressure–flow interactions during valve opening and closure (Sacks et al., 2009; Patrick et al., 2011). In addition, the coupling between ventricular mechanics, vascular response, and valve dynamics is often treated in a partially decoupled manner, limiting the ability to reproduce pressure, flow, and volume waveforms with consistent phase relationships (Antonini-Canterin et al., 2013; Menon et al., 2013; Sharma and Chen, 2025). Furthermore, systematic evaluation of parameter sensitivity under combined physiological and pathological conditions remains limited. As a result, the relative contributions of myocardial properties, vascular compliance, and geometric valve alterations to global hemodynamic behavior are not clearly quantified (Shen et al., 2026; Goktas et al., 2015).

To address these limitations, a closed-loop lumped-parameter cardiovascular model was developed to capture the coupled interaction between cardiac chambers, vascular compartments, and valve dynamics within a unified framework. The model incorporated time-varying elastance to represent myocardial mechanics, resistance, compliance, and inertance elements for vascular behavior, and pressure-driven nonlinear valve flow relations. In addition, valvular stenosis and ageing-related changes in ventricular elastance and arterial compliance were integrated using physiologically consistent parameters. Unlike previous studies, which typically examined these factors independently, the present work evaluates their combined and interacting effects on pressure, flow, and volume dynamics. A systematic sensitivity analysis was performed to quantify the relative contributions of arterial compliance, ventricular stiffness, and stenosis severity to key hemodynamic outputs. This approach provides a mechanistic understanding of how structural and functional alterations jointly influence cardiovascular performance.

The present study aimed to quantify the relative roles of myocardial, vascular, and geometric factors in governing cardiovascular function using the proposed framework. Model predictions were analyzed using time-resolved hemodynamic waveforms, pressure–volume relationships, and sensitivity trends. The results provided a consistent representation of ventricular–arterial coupling and valve-mediated flow dynamics. In addition, the framework offered a computationally efficient approach for identifying dominant mechanisms influencing cardiovascular performance. These findings contribute to improved interpretation of diagnostic indicators and provide a foundation for future development of patient-specific cardiovascular models.

## 2 Materials and methods

A closed-loop lumped-parameter cardiovascular model was developed to investigate the coupled interaction among ventricular mechanics, valve dynamics, and systemic–pulmonary circulation under physiological and pathological conditions. The framework integrated time-varying elastance models for the cardiac chambers with resistance–compliance–inertance (R–C–L) representations of the vascular system and pressure-driven nonlinear valve flow relations. Similar compartment-coupling strategies have been widely applied in cardiovascular modeling studies to reproduce global hemodynamic behavior efficiently while preserving essential physiological interactions (Munneke et al., 2022; Scarsoglio et al., 2014; Korakianitis and Shi, 2006; Mynard et al., 2012). In the present study, the model was extended to incorporate aortic stenosis and age-related alterations in ventricular stiffness and vascular compliance to evaluate their combined influence on cardiovascular hemodynamics. The governing equations describing chamber pressure, vascular flow, valve dynamics, and compartment coupling were solved simultaneously to obtain time-resolved pressure, flow, and volume waveforms throughout the cardiac cycle. The governing relationships used for vascular resistance, compliance, inertance, chamber pressure, chamber volume conservation, and valve flow were described by Equations 1–7.

### 2.1 Lumped parameter representation of the cardiovascular system

**FIGURE 1:**
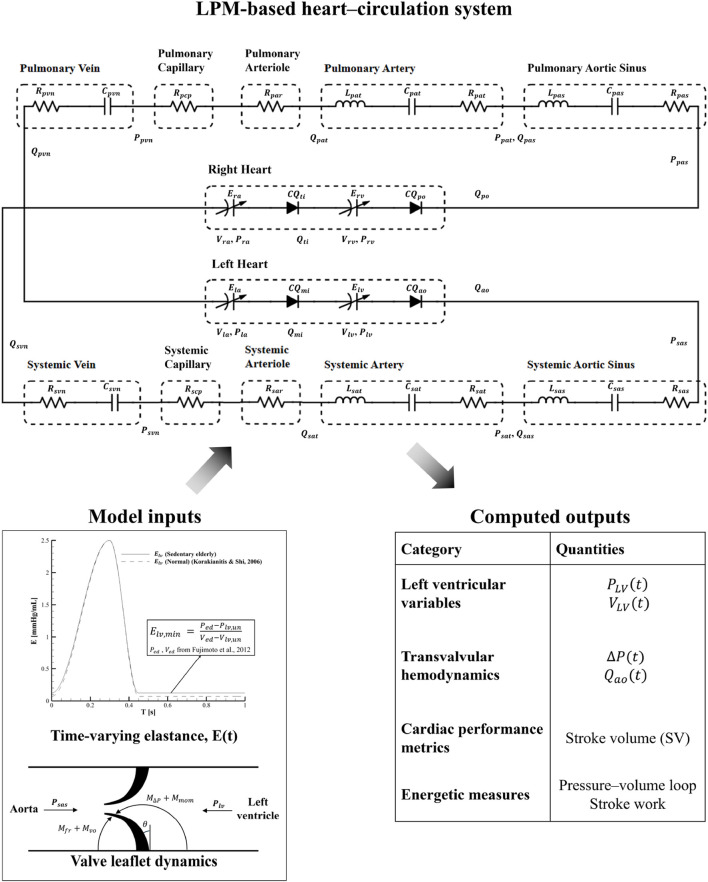
Lumped parameter model of the closed-loop cardiovascular system illustrating the coupling between cardiac chambers, valve dynamics, and vascular compartments. The left and right hearts are represented by time-varying elastance models for the atria and ventricles, where chamber pressures depend on instantaneous volume and elastance. Cardiac valves (mitral, aortic, tricuspid, and pulmonary) regulate flow between adjacent compartments and are modeled using pressure-driven nonlinear flow relations with a dynamically varying effective opening area. The pulmonary and systemic circulations are represented using interconnected resistance (R), compliance (C), and inertance (L) elements, corresponding to viscous losses, vascular elasticity, and blood inertia, respectively. Arterial segments incorporate R–L–C configurations to capture pulsatile flow dynamics and pressure wave propagation, whereas venous and capillary compartments are modeled primarily using R–C elements due to negligible inertial effects. Pressure (P), flow rate (Q), and volume (V) variables are defined at each compartment, ensuring continuity and full coupling across the network. The closed-loop structure enables simulation of the integrated interactions among ventricular mechanics, valve operation, and vascular response throughout the cardiac cycle.

The cardiovascular system was represented by a closed-loop lumped-parameter model, as illustrated in Figure 1. The model consisted of four cardiac chambers (left atrium, left ventricle, right atrium, and right ventricle) coupled with the pulmonary and systemic circulations through pressure-driven flow interactions. Each compartment shown in Figure 1 was associated with pressure (P), flow rate (Q), and volume (V) variables, ensuring complete coupling of the cardiovascular network. The global hemodynamic behavior was governed by ordinary differential equations derived from conservation of mass and momentum. The complete mathematical formulation of all cardiac chambers and vascular segments was provided in the Supplementary Material.

### 2.2 Vascular segment modeling

As shown in Figure 1, the pulmonary and systemic circulations were represented using resistance (R), compliance (C), and inertance (L) elements, corresponding to viscous losses, elastic storage, and inertial effects, respectively.

$$\Delta P_{R}=RQ$$ (1)

where Q denoted volumetric blood flow rate. Vascular compliance was modeled as a capacitive element relating pressure and stored volume according to

$$C=\frac{dV}{dP}$$ (2)

which yielded the pressure evolution equation

$$\frac{dP}{dt}=\frac{1}{C}\left(Q_{in}-Q_{out}\right)$$ (3)

Inertial effects associated with pulsatile flow were modeled using inductive elements, where the pressure drop due to blood inertia was expressed as

$$\Delta P_{L}=L\frac{dQ}{dt}$$ (4)

Pulmonary and systemic arterial segments were modeled using R–L–C configurations to capture wave propagation and damping effects, whereas venous and capillary segments were modeled primarily using R–C elements due to negligible inertial contributions, is summarized in Supplementary Tables 4, 5.

### 2.3 Pulmonary and systemic circulation

The pulmonary and systemic circuits illustrated in Figure 1 were arranged in series and connected through the right and left heart, respectively. The pulmonary circulation included arteries, arterioles, capillaries, and veins, with resistance concentrated in the microcirculation and compliance distributed across arterial and venous segments. The systemic circulation consisted of the aorta, systemic arteries, peripheral vessels, and systemic veins, as shown in Figure 1. Parameter values were selected from previously published cardiovascular modeling studies and physiological data reported in the literature to ensure physiologically realistic pressure and flow conditions. The selected values were maintained within physiologically acceptable ranges for both healthy and pathological cardiovascular states.

### 2.4 Cardiac chamber modeling

The left and right hearts were modeled using the time-varying elastance formalism. Atrial and ventricular pressures were defined as

$$P\left(t\right)=E\left(t\right)\left[V\left(t\right)-Vo\right]$$ (5)

where E(t) represented the time-varying elastance, V(t) denoted chamber volume, and V_0_ was the unstressed volume. Elastance functions were prescribed as periodic waveforms synchronized with the cardiac cycle to represent systolic contraction and diastolic relaxation.

Chamber volume dynamics were governed by mass conservation, expressed as

$$\frac{dV}{dt}=Q_{in}-Q_{out}$$ (6)

Initial conditions for pressures and volumes were prescribed based on physiological values and were listed in Supplementary Table 2.

### 2.5 Valve dynamics

The cardiac valves shown in Figure 1 (mitral, aortic, tricuspid, and pulmonary) regulated flow between adjacent compartments. Valve behavior was modeled using pressure-driven nonlinear flow relations with a dynamically varying effective opening area.

The transvalvular flow rate was defined as

$$Q_{valve}=\left\{\begin{array}{l} \frac{P_{up}-P_{down}\;}{R_{valve}}\\ 0, \end{array}\right.\frac{P_{up}>P_{down}}{P_{up}\leq P_{down}}$$ (7)

where P_up_ and P_down_ denoted upstream and downstream pressures, respectively. This formulation enforced unidirectional flow and prevented regurgitation. Valve parameters, including flow coefficients and dynamic response terms, were provided in Supplementary Table 6. This formulation enabled the representation of valve opening, closure, and transient flow reversal. Similar valve-modeling strategies have been reported in previous lumped-parameter cardiovascular studies, including the work of Mynard et al. (Mynard et al., 2012), where dynamic valve formulations were used to represent physiologically realistic transvalvular flow behavior under normal and pathological conditions. In the present study, aortic stenosis was represented through modification of aortic valve-related parameters governing transvalvular resistance and flow dynamics. Additional variations in ventricular stiffness and vascular compliance were introduced separately to represent age-related cardiovascular alterations that may coexist with aortic stenosis in elderly populations.

### 2.6 Model coupling and numerical solution

All compartments were coupled through pressure and flow continuity conditions, forming a closed-loop system of ordinary differential equations. The resulting system was solved numerically using time-marching integration with physiologically realistic initial conditions. Periodic steady-state solutions were obtained after several cardiac cycles. This modeling framework enabled efficient simulation of cardiovascular dynamics while preserving essential physiological mechanisms governing cardiac–vascular interaction. The formulation allowed direct modification of cardiac elastance, vascular resistance, and compliance to investigate both normal and pathological hemodynamic conditions. The full set of coupled governing equations was provided in the Supplementary Material.

### 2.7 Numerical implementation

The system of nonlinear ordinary differential equations was solved using a variable-step, implicit solver (ode15s, MATLAB), suitable for stiff cardiovascular systems. The time step was automatically adjusted to ensure numerical stability and accuracy. Simulations were performed over multiple cardiac cycles until periodic steady-state conditions were achieved. Convergence was assessed using the normalized L^2^-norm error between successive cardiac cycles, with a convergence threshold of 10^−2^. Under all simulated conditions, convergence was achieved after approximately 5–10 cardiac cycles. Results were extracted from the final cardiac cycle.

### 2.8 Model validation and parametric analysis

Model predictions at key locations shown in Figure 1, including the left ventricle, aorta, and valves, were compared with the reference model of Korakianitis and Shi (2006) (Korakianitis and Shi, 2006). This reference model was selected because it is a widely established benchmark in lumped-parameter cardiovascular simulations and provides physiologically consistent pressure, flow, and valve-dynamics behavior. The comparison was performed to confirm that the present 0D closed-loop model reproduced the fundamental hemodynamic characteristics of ventricular–vascular interaction and valve-mediated flow dynamics. Since lumped-parameter cardiovascular models are simplified 0D representations, the evaluation focused primarily on physiologically relevant hemodynamic indices, including systolic and diastolic pressures, ventricular pressure–volume behavior, peak flow rates, stroke volume, ejection characteristics, and transvalvular pressure differences, rather than exact reproduction of every transient waveform feature. Therefore, the present comparison should be regarded as a benchmark verification and physiological consistency assessment rather than a direct clinical validation against patient-specific data. The reference model was used to assess the implemented governing equations and coupled hemodynamic behavior, rather than for direct parameter optimization or waveform fitting.

### 2.9 Reproducibility

All governing equations, parameter values, initial conditions, and model structure required to reproduce the simulations were provided in the Supplementary Material. The modular formulation allows direct parameter modification for further analysis.

## 3 Results and discussion

### 3.1 Time-resolved hemodynamic response of the left ventricle and validation against reference model

**FIGURE 2:**
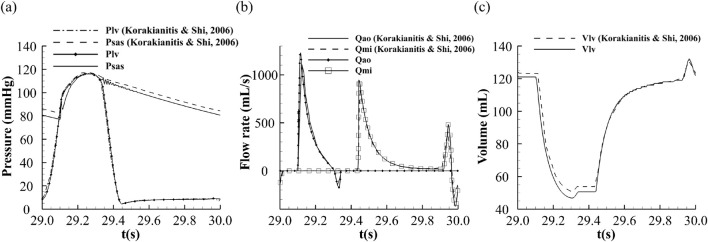
Time-resolved hemodynamic waveforms of the left heart over one complete cardiac cycle and comparison with the reference model of Korakianitis and Shi (2006). **(a)** Left ventricular pressure (P_lv_) and systemic aortic sinus (P_sas_), demonstrating isovolumetric contraction, systolic ejection, and diastolic decay with clear identification of the dicrotic notch. **(b)** Aortic (Q_ao_) and mitral (Q_mi_) flow rates, illustrating systolic ejection through the aortic valve and biphasic diastolic filling (E-wave and A-wave) through the mitral valve. **(c)** Left ventricular volume variation, showing end-diastolic and end-systolic volumes, stroke volume, and distinct isovolumetric phases. Solid lines represent the present model, and dashed lines indicate the reference data.

The temporal evolution of left ventricular pressure, systemic arterial pressure, transvalvular flow rates, and ventricular volume during one cardiac cycle is presented in Figure 2. These variables collectively describe the mechanical pumping function of the left ventricle and the interaction between myocardial contraction, valve dynamics, and the systemic circulation. In physiological conditions, the cardiac cycle consists of four phases: isovolumetric contraction, ejection, isovolumetric relaxation, and filling. A specific relationship between pressure, flow, and volume characterizes each phase. Therefore, simultaneous evaluation of these variables was used to assess the accuracy of the implemented lumped-parameter model. The predicted results under healthy physiological conditions were compared with the reference healthy cardiovascular model of Korakianitis and Shi (2006) to verify physiological consistency and numerical stability. The pressure waveforms shown in Figure 2a represent the dynamic response of the left ventricle and the systemic arterial system. At the onset of systole, the left ventricular pressure increased rapidly while the ventricular volume remained nearly constant, indicating the isovolumetric contraction phase. During this phase, both the mitral and aortic valves remained closed, and the pressure rise was governed by the time-varying elastance of the ventricular myocardium, which defines the chamber’s pressure–volume relationship. The peak systolic ventricular pressure reached approximately 115–120 mmHg, which lies within the normal physiological range. When the ventricular pressure exceeded the systemic arterial pressure, the aortic valve opened and the ejection phase began. During early ejection, the ventricular and arterial pressures followed each other closely, indicating proper ventricular–arterial coupling. As systole progressed, both pressures gradually decreased due to reduced ventricular elastance and declining flow rate. The arterial pressure waveform showed a rapid systolic rise followed by a gradual diastolic decay, governed by arterial compliance and peripheral resistance. A distinct dicrotic notch was observed at the instant of valve closure, representing flow deceleration and elastic recoil of the aorta. Small oscillatory ripples were observed in the systemic arterial pressure waveform after aortic valve closure compared with the smoother waveform reported in the reference model. These oscillations originated from the coupled resistance–compliance–inertance dynamics of the systemic arterial compartment. After valve closure, the aortic inflow rapidly decreased to zero, while the downstream arterial flow response remained governed by vascular inertance, resistance, and compliance. The resulting transient oscillatory behavior in the systemic arterial flow was subsequently transferred to the pressure waveform through the pressure–flow coupling relation. Therefore, the observed ripples were interpreted as the local transient response of the lumped arterial compartment rather than numerical instability. Despite these small oscillations, the overall pressure magnitude, waveform morphology, and temporal behavior remained consistent with the reference model. The transvalvular flow rate profiles presented in Figure 2b provide further insight into valve-mediated hemodynamics. The aortic flow increased rapidly after valve opening and reached a peak exceeding 1,000 mL/s during early systole. This peak corresponded to the maximum pressure gradient between the ventricle and the aorta. As the pressure difference decreased during late systole, the flow rate declined gradually. Near the end of ejection, a brief negative flow was observed, indicating flow reversal associated with valve closure and inertial effects of the blood. This feature confirmed that the model accounted for valve dynamics rather than assuming ideal one-way flow behavior. The mitral inflow waveform exhibited a characteristic biphasic pattern consisting of an early filling peak (E-wave) followed by a late filling peak due to atrial contraction (A-wave). These features arise from the interaction among atrial pressure, ventricular relaxation, and valve-opening dynamics, as reported in advanced cardiovascular models. The timing and magnitude of these peaks indicated realistic diastolic filling behavior. The ventricular volume variation shown in Figure 2c reflects the combined effect of inflow and outflow throughout the cardiac cycle. At end-diastole, the ventricular volume was approximately 120 mL, representing the preload condition. During isovolumetric contraction, the volume remained constant while pressure increased. Once the aortic valve opened, rapid ejection resulted in a significant decrease in volume, reaching an end-systolic volume of approximately 50 mL. This resulted in a stroke volume of about 70 mL, which is consistent with normal physiological values reported in the reference model. After ejection, the volume remained nearly constant during isovolumetric relaxation until the mitral valve opened. Subsequently, rapid early filling increased the ventricular volume, followed by a slower filling phase and a final increment due to atrial contraction. The close agreement of these values with the reference model confirmed proper coupling between ventricular elastance, valve dynamics, and vascular resistance. The combined interpretation of pressure, flow, and volume demonstrated correct phase synchronization throughout the cardiac cycle. Valve opening and closure occurred when the corresponding pressure gradients changed sign, and zero-flow intervals coincided with isovolumetric phases. Peak flow rates occurred at times of maximum pressure difference between interacting compartments. These results showed strong agreement with the established cardiovascular model of Korakianitis and Shi (2006), confirming that the present formulation accurately reproduced the fundamental features of ventricular–arterial interaction and cardiac pumping mechanics. This agreement validated the governing equations and parameter selection, providing a reliable basis for further parametric and pathological analysis.

### 3.2 Hemodynamic characteristics of aortic valve dynamics and ventricular pumping behavior

**FIGURE 3:**
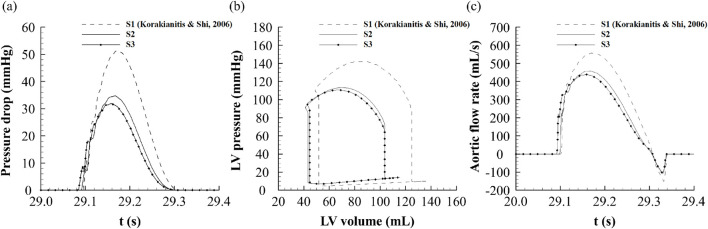
Hemodynamic characteristics of aortic valve dynamics and ventricular pumping behavior under three simulated conditions: aortic stenosis (AS), aortic stenosis with increased left ventricular stiffness (AS + LV stiffness), and aortic stenosis with reduced vascular compliance (AS + VC↓). **(a)** Temporal variation of the transvalvular pressure difference during the ejection phase, defined as $\Delta P\left(t\right)=P_{lv}\left(t\right)-P_{sas}\left(t\right)$ , evaluated only when the aortic valve flow $Q_{ao}\left(t\right)$ > 0. This parameter represents the instantaneous pressure load across the aortic valve that the left ventricle must overcome during blood ejection. **(b)** Left ventricular pressure–volume (PV) loop illustrating the relationship between ventricular pressure and volume over one complete cardiac cycle. The loop describes the sequential phases of ventricular filling, isovolumetric contraction, ejection, and isovolumetric relaxation, and reflects the mechanical work performed by the ventricle. **(c)** Aortic valve flow waveform $Q_{ao}\left(t\right)$ showing the instantaneous blood flow rate through the aortic valve during systole. The waveform indicates the onset of valve opening, peak ejection flow, and the deceleration of flow associated with valve closure dynamics.

The mechanical performance of the left ventricle and the associated aortic valve dynamics were evaluated through the transvalvular pressure difference, the ventricular pressure–volume (PV) relationship, and the aortic valve flow waveform. These parameters describe the interaction between ventricular contraction, valve opening, and blood ejection into the systemic circulation. Under physiological conditions, the aortic valve opens when the left ventricular pressure exceeds the systemic arterial pressure, enabling forward flow. Therefore, the pressure gradient across the valve, the ventricular PV trajectory, and the resulting flow waveform collectively characterizes ventricular–arterial coupling and ejection efficiency. Figure 3a shows the temporal variation of the transvalvular pressure difference during the ejection phase. The pressure difference was defined as $\Delta P\left(t\right)=P_{lv}\left(t\right)-P_{sas}\left(t\right)$ *,* and was evaluated only when the aortic valve flow Q_ao(_t) was positive, corresponding to the valve-open condition. It was observed that the pressure difference increased rapidly at the onset of ejection, reached a peak during early systole, and then gradually decreased as ventricular pressure declined. For the reference case (S1) (Korakianitis and Shi, 2006), the peak pressure difference was 50 mmHg, whereas reduced values of 35 mmHg (S2) and 30 mmHg (S3) were observed. Differences in peak magnitude were observed among the simulated cases, including aortic stenosis, increased ventricular stiffness, and combined pathological conditions. A higher pressure difference indicated increased resistance to flow through the valve and a greater mechanical load on the ventricle. This behavior was consistent with the nonlinear pressure–flow relationship across the valve, in which the pressure gradient increases with increasing flow resistance and reduced effective valve area. The ventricular PV relationship over one cardiac cycle is presented in Figure 3b. The PV loop represents the mechanical behavior of the ventricle during filling, isovolumetric contraction, ejection, and isovolumetric relaxation. During filling, the ventricular volume increased while pressure remained low due to chamber compliance. This was followed by isovolumetric contraction, during which pressure increased rapidly while volume remained nearly constant. Once the ventricular pressure exceeded arterial pressure, the aortic valve opened and the ejection phase began, reducing volume at elevated pressure. After ejection, isovolumetric relaxation occurred, during which pressure decreased rapidly before the next filling phase started. The end-diastolic volume was approximately 120–130 mL, while the end-systolic volume ranged between 50–70 mL across the cases, resulting in a stroke volume of 50–70 mL. The peak ventricular pressure reached 120–140 mmHg. Variations in the size and shape of the PV loops across conditions reflected changes in ventricular stiffness and loading conditions, which directly influenced stroke volume and cardiac work. This behavior followed the elastance-based ventricular model, in which pressure depends on time-varying elastance and chamber volume. The instantaneous aortic valve flow rate waveform is shown in Figure 3c. The flow increased rapidly after valve opening, reaching a peak during early systole, when the pressure gradient between the ventricle and the aorta was at its maximum. Subsequently, the flow decreased gradually as ventricular contraction weakened. The peak flow rate ranged from 500 to 600 mL/s in the reference case, whereas reduced peak values of 400–500 mL/s were observed under altered conditions. Near the end of systole, a brief negative flow was observed, indicating a short period of flow reversal associated with valve closure and deceleration of blood in the aorta. The magnitude of this reverse flow was small (−50 to −100 mL/s), confirming physiologically realistic valve-closure dynamics. This feature represents regurgitant flow arising from valve dynamics and pressure reversal during late systole, as reported in advanced cardiovascular models. Differences in peak flow and waveform morphology across the simulated cases indicated variations in ejection efficiency and valve-opening characteristics. Compared with the baseline waveform presented in Figure 2b, the flow waveforms in Figure 3c exhibited altered systolic peak behavior due to pathological alterations in valve resistance, ventricular stiffness, and vascular loading conditions. Although stroke volume and left ventricular ejection duration remained relatively similar across cases, the instantaneous acceleration and deceleration characteristics of systolic flow were strongly influenced by altered transvalvular pressure gradients and ventricular–vascular interactions. These effects modified the peak morphology and temporal profile of the aortic flow waveform while preserving the overall volumetric output. The time integral of the flow waveform yielded a stroke volume consistent with the PV loop estimation (∼60 mL). Overall, the combined analysis of the transvalvular pressure difference, PV loop, and flow waveform demonstrated that the model captured key physiological features of ventricular ejection and valve-mediated flow dynamics. The results showed consistent agreement with established cardiovascular behavior, including nonlinear pressure gradients, characteristic PV loop morphology, and realistic systolic flow patterns. These findings confirmed that the model provided a reliable representation of ventricular pumping mechanics under both normal and pathological conditions.

The quantitative comparison of key hemodynamic outputs across the healthy baseline and pathological conditions is summarized in Table 1. The healthy c ase served as the reference condition, whereas S1 represented aortic stenosis, S2 represented aortic stenosis with increased left ventricular stiffness, and S3 represented aortic stenosis with increased ventricular stiffness and reduced arterial compliance.

**TABLE 1:** Summary of SV and PP under different pathological conditions. S1 represents aortic stenosis (AS), S2 includes AS with increased left ventricular stiffness, and S3 combines AS, increased ventricular stiffness, and reduced arterial compliance.

Scenario	SV (mL/beat)	PP (mmHg)
Healthy baseline	68.7 ± 17	42.8 ± 9.6
S1 aortic stenosis (AS)	76.16	36.28
S2 AS + increased LV stiffness	62.79	35.07
S3 AS + LV stiffness + reduced compliance	60.98	40.64

### 3.3 Sensitivity of hemodynamic outputs to cardiovascular parameters

**FIGURE 4:**
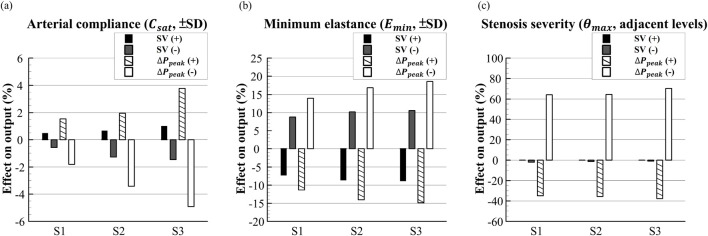
Sensitivity of model outputs to variations in key cardiovascular parameters under three operating > conditions (S1–S3). **(a)** Effect of arterial compliance (C_sat_, ±SD), **(b)** effect of minimum elastance (E_min_, ±SD), and **(c)** effect of stenosis severity (θ_max_, adjacent levels) on stroke volume (SV) and peak pressure difference (ΔP_peak_). Positive (+) and negative (−) perturbations indicate increases and decreases, respectively, in the corresponding input parameters. The results showed that arterial compliance had a relatively small influence on both outputs; minimum elastance produced moderate changes, with increasing sensitivity from S1 to S3; and stenosis severity had a dominant effect on ΔP_peak_ with minimal impact on SV.

The cardiovascular system is governed by a coupled interaction between myocardial mechanics, vascular properties, and geometric constraints imposed by pathological conditions such as stenosis. In lumped-parameter cardiovascular modeling, arterial compliance (C_sat_) quantified the arterial system’s ability to store blood during systole and release it during diastole. At the same time, minimum elastance (E_min_) described the passive stiffness of the ventricle during diastolic filling. In addition, stenosis severity (θ_max_) quantified the geometric restriction to flow through the valve. These parameters directly influenced stroke volume (SV) and the transvalvular pressure gradient (ΔP_peak_), both key indicators of cardiac performance. Such relationships were consistent with established cardiovascular modeling approaches, in which ventricular elastance governs pressure–volume behavior and vascular compliance regulates pressure buffering. A systematic sensitivity analysis was performed to quantify the relative influence of these parameters under different physiological > conditions (S1–S3), and the results are presented in Figure 4. It was observed that arterial compliance had a relatively weak influence on both SV and ΔP_peak,_ as shown in Figure 4a. The variation in SV remained within approximately ±1%, while ΔP_peak_ varied within ±2%–5%. This limited sensitivity indicated that moderate changes in arterial compliance did not significantly alter ventricular ejection or pressure generation. This behavior was consistent with the role of compliant arteries in damping pressure oscillations and stabilizing flow, as described in the resistance–compliance framework of the systemic circulation. Therefore, arterial compliance primarily served as a buffer rather than a dominant driver of hemodynamic output. In contrast, minimum elastance (E_min_) exhibited a significantly stronger influence on both SV and ΔP_peak,_ as illustrated in Figure 4b. The variation in SV was approximately ±8%–10%, while ΔP_peak_ changed by ±12%–18%, demonstrating a higher sensitivity of the system to ventricular diastolic properties. An increase in E_min_, corresponding to increased ventricular stiffness, reduced ventricular compliance, and limited end-diastolic filling. As a result, stroke volume decreased, while pressure increased due to reduced chamber distensibility. Conversely, a decrease in E_min_ enhanced ventricular filling, resulting in increased SV and reduced pressure gradients. This behavior directly followed from the elastance-based pressure–volume relationship used to describe ventricular mechanics. Furthermore, the magnitude of sensitivity increased progressively from S1 to S3, indicating that under altered or pathological conditions, the cardiovascular system became more sensitive to variations in myocardial relaxation properties. This trend highlighted the critical importance of diastolic function in regulating cardiac output, particularly in compromised physiological states. The most pronounced effect was observed for stenosis severity (θ_max_), which showed a negligible influence on SV but a dominant impact on ΔP_peak,_ as presented in Figure 4c. Stroke volume remained nearly unchanged across all conditions, suggesting that the cardiovascular system compensated to preserve volumetric output despite geometric obstruction. However, ΔP_peak_ exhibited large asymmetric variations, ranging from approximately −35% to +70%. This strong sensitivity arose from the nonlinear relationship between pressure drop and flow through a restricted orifice, where the pressure gradient scales with the square root of the flow and is strongly dependent on the effective valve opening area. As a result, even small increases in stenosis severity led to disproportionately large increases in the pressure gradient. The observed asymmetry was attributed to the use of adjacent severity levels rather than symmetric perturbations, reflecting the nonlinear progression of stenotic disease. Overall, the comparative analysis revealed a clear hierarchy in parameter influence. Arterial compliance had a minor stabilizing effect, minimum elastance had a moderate and condition-dependent influence, and stenosis severity dominated pressure-related responses. These findings indicated that vascular properties primarily governed pressure buffering, myocardial stiffness controlled volumetric output, and geometric obstruction dictated pressure loading conditions. This distinction has important clinical implications: pressure-based diagnostic metrics are highly sensitive to stenosis progression, whereas volume-based metrics are more closely associated with ventricular function. The results demonstrated that the present model was capable of capturing physiologically consistent sensitivity trends, in agreement with established cardiovascular dynamics. Such analysis is essential for improving model reliability, guiding parameter estimation, and supporting patient-specific simulations for diagnostic and therapeutic applications.

## 4 Conclusion

An LPM of the cardiovascular system was implemented to simulate the dynamic interaction between left ventricular mechanics, valve behavior, and systemic circulation. The model successfully reproduced physiologically consistent pressure, flow, and volume waveforms over the cardiac cycle, and the results showed strong agreement with the reference model. This agreement confirmed the validity of the governing equations, elastance formulation, and valve dynamics representation. The sensitivity analysis demonstrated that arterial compliance had a limited effect on primary hemodynamic outputs, suggesting a secondary buffering role. Minimum elastance significantly influenced both stroke volume and pressure generation, underscoring the importance of ventricular diastolic properties in regulating cardiac performance. Stenosis severity had the strongest effect on the transvalvular pressure gradient, confirming that geometric restriction of the valve is the dominant factor governing pressure overload. The results further showed that system sensitivity increased under altered physiological conditions, indicating enhanced dependence on myocardial and valve parameters in pathological states. The study provided a clear quantitative ranking of parameter influence and highlighted the distinct roles of vascular, myocardial, and geometric factors in cardiovascular dynamics. These results have significant implications for the interpretation of diagnostic metrics: pressure-based indicators were highly sensitive to stenosis progression. At the same time, volume-based measures were more closely related to ventricular function. The present model provides a reliable, computationally efficient framework for analyzing cardiovascular behavior and can be extended for patient-specific simulations, disease modeling, and therapeutic planning.

## Data Availability

The original contributions presented in the study are included in the article/Supplementary Material, further inquiries can be directed to the corresponding author.

## Nomenclature

A: sectional area
C: compliance
CQ: flow coefficient
E: elastance
I: inertial moment of rotating
K,k: coefficient
L: Inertance
P: pressure
Q: flow rate
R: resistance
T: time; heart period
V: volume
θ: rotating angle of valve leaflet
$K_{p}$: pressure-related force coefficient in valve dynamics
$K_{f}$: friction-related coefficient due to surrounding tissue
$K_{b}$: blood momentum-related coefficient
$K_{v}$: vortex-induced force coefficient
$CQ_{mi}$: mitral valve flow coefficient
$CQ_{\text{ao}}$: aortic valve flow coefficient
$CQ_{\text{ti}}$: tricuspid valve flow coefficient
$CQ_{\text{po}}$: pulmonary valve flow coefficient
e: elastance activation function
$e_{a}$: atrial activation function
$e_{v}$: ventricular activation function
0: initial value; offset value
un: unstressed
ao: aortic valve
mi: mitral valve
ti: tricuspid valve
po: pulmonary valve
la: left atrium
lv: left ventricle
ra: right atrium
rv: right ventricle
sas: systemic aortic sinus
sat: systemic artery
sar: systemic arterioles
scp: systemic capillary
svn: systemic vein
pas: pulmonary arterial sinus
pat: pulmonary artery
par: pulmonary arterioles
pcp: pulmonary capillary
pvn: pulmonary vein
max: maximum value
min: minimum value
fr: frictional effect on valve dynamics
vo: vortex effect on valve dynamics
vo: vortex effect on valve dynamics
ss: shear stress effect on valve dynamics
ΔP: pressure difference across valve
