# Supplementary material for: Integrated lumped parameter modeling of cardiac–vascular interaction with valve dynamics and ventricular pressure–volume
Source: Front Bioeng Biotechnol. 2026 Jul 13;14:1841734. doi: 10.3389/fbioe.2026.1841734 (PMC13402562; doi:10.3389/fbioe.2026.1841734)
Supplement: Supplementary file 1 [file Supplementaryfile1.docx]

Supplementary Material

Integrated Lumped Parameter Modeling of Cardiac–Vascular Interaction with Valve Dynamics and Ventricular Pressure–Volume

Prashant Kishor Sharma ^1†^, Wen Kai Yang ^1†^, and Chia-Yuan Chen ^1*^

^1^ Department of Mechanical Engineering, National Cheng Kung University, Tainan, 701, Taiwan

^†^ Contributed equally

*** Correspondence:**Corresponding Author: Chia Yuan Chen
chiayuac@mail.ncku.edu.tw

**Supplementary file contains:**

**S1. Mathematical Model Overview**

This supplementary material provides the complete mathematical formulation of the lumped parameter cardiovascular model used in this study. The governing equations for all cardiac chambers, vascular networks, and valve dynamics are presented. In addition, the numerical implementation, initial conditions, and parameter values are listed to ensure full reproducibility of the simulations. The cardiovascular system was represented as a closed-loop network, with each compartment described using pressure–volume relationships and flow continuity principles based on conservation of mass and momentum [1].

**S2. Governing Equations and Numerical Scheme**

The system consisted of a set of coupled ordinary differential equations describing the temporal evolution of pressure, flow rate, and volume in each compartment. The model was solved using a stiff ordinary differential equation solver (ode15s). Simulations were performed over multiple cardiac cycles until periodic steady-state behavior was achieved.

**S3. Cardiac Chamber Modeling**

**S3.1. Left Atrium**

The left atrium was modeled using a time-varying elastance formulation, in which pressure was related to chamber volume and an activation function.

| $\frac{dV_{la}}{dt}=Q_{pvn}-Q_{mi}$ | (1) |
| --- | --- |
| $P_{la}=P_{la,un}+E_{la}\left( V_{la}-V_{la,un} \right)$ | (2) |
| $Q_{mi}=\left\{ \begin{matrix} C_{Qmi}AR_{mi}\sqrt{P_{la}-P_{lv}}, & P_{la}\geq P_{lv} \\ -C_{Qmi}AR_{mi}\sqrt{P_{lv}-P_{la}}, & P_{la}<P_{lv} \end{matrix} \right.$ | (3) |
| $E_{la}\left( t \right)=E_{la,min}+\frac{E_{la,max}-E_{la,min}}{2}e_{a}\left( t \right)$ | (4) |
| $e_{a}\left( t \right)=\left\{ \begin{matrix} 0, & 0\leq t\leq T_{ac} \\ 1-cos\left( \frac{t-T_{ac}}{RR-T_{ac}}2\pi\right), & T_{ac}<t\leq RR \end{matrix} \right.$ | (5) |
| $AR_{mi}=\frac{\left( 1-cos\theta_{mi} \right)^{2}}{\left( 1-cos\theta_{mi,max} \right)^{2}}$ | (6) |
| $\frac{d^{2}\theta_{mi}}{dt^{2}}=\left\{ \begin{matrix} \left( P_{la}-P_{lv} \right)K_{p,mi}\cos\theta_{mi}-K_{f,mi}\frac{d\theta_{mi}}{dt}+K_{b,mi}Q_{mi}\cos\theta_{mi}-K_{v,mi}Q_{mi}\sin\left( 2\theta_{mi} \right), & Q_{mi}\geq0 \\ \left( P_{la}-P_{lv} \right)K_{p,mi}\cos\theta_{mi}-K_{f,mi}\frac{d\theta_{mi}}{dt}+K_{b,mi}Q_{mi}\cos\theta_{mi}, & Q_{mi}<0 \end{matrix} \right.$ | (7) |

**S3.2. Left Ventricle**

The left ventricle was represented using a nonlinear time-varying elastance model to capture systolic contraction and diastolic relaxation.

| $\frac{dV_{lv}}{dt}=Q_{mi}-Q_{ao}$ | (8) |
| --- | --- |
| $P_{lv}=P_{lv,un}+E_{lv}\left( V_{lv}-V_{lv,un} \right)$ | (9) |
| $Q_{ao}=\left\{ \begin{matrix} C_{Qao}AR_{ao}\sqrt{P_{lv}-P_{sas}}, & P_{lv}\geq P_{sas} \\ -C_{Qao}AR_{ao}\sqrt{P_{sas}-P_{lv}}, & P_{lv}<P_{sas} \end{matrix} \right.$ | (10) |
| $E_{lv}\left( t \right)=E_{lv,min}+\frac{E_{lv,max}-E_{lv,min}}{2}e_{v}\left( t \right)$ | (11) |
| $e_{v}\left( t \right)=\left\{ \begin{matrix} 1-cos\left( \frac{t}{T_{me}}\pi\right), & 0\leq t<T_{me} \\ 1+cos\left( \frac{t-T_{me}}{T_{ce}-T_{me}}\pi\right), & T_{me}\leq t<T_{ce} \\ 0, & T_{ce}\leq t\leq RR \end{matrix} \right.$ | (12) |
| $AR_{ao}=\frac{\left( 1-cos\theta_{ao} \right)^{2}}{\left( 1-cos\theta_{ao,max} \right)^{2}}$ | (13) |
| $\frac{d^{2}\theta_{ao}}{dt^{2}}=\left\{ \begin{matrix} \left( P_{lv}-P_{sas} \right)K_{p,ao}\cos\theta_{ao}-K_{f,ao}\frac{d\theta_{ao}}{dt}+K_{b,ao}Q_{ao}\cos\theta_{ao}-K_{v,ao}Q_{ao}\sin\left( 2\theta_{ao} \right), & Q_{ao}\geq0 \\ \left( P_{lv}-P_{sas} \right)K_{p,ao}\cos\theta_{ao}-K_{f,ao}\frac{d\theta_{ao}}{dt}+K_{b,ao}Q_{ao}\cos\theta_{ao}, & Q_{ao}<0 \end{matrix} \right.$ | (14) |

**S3.3. Right Atrium**

The right atrium was described using a similar elastance-based formulation, accounting for venous return and atrial contraction:

| $\frac{dV_{ra}}{dt}=Q_{svn}-Q_{ti}$ | (15) |
| --- | --- |
| $P_{ra}=P_{ra,un}+E_{ra}\left( V_{ra}-V_{ra,un} \right)$ | (16) |
| $Q_{ti}=\left\{ \begin{matrix} C_{Qti}AR_{ti}\sqrt{P_{ra}-P_{rv}}, & P_{ra}\geq P_{rv} \\ -C_{Qti}AR_{ti}\sqrt{P_{rv}-P_{ra}}, & P_{ra}<P_{rv} \end{matrix} \right.$ | (17) |
| $E_{ra}\left( t \right)=E_{ra,min}+\frac{E_{ra,max}-E_{ra,min}}{2}e_{a}\left( t \right)$ | (18) |
| $AR_{ti}=\frac{\left( 1-cos\theta_{ti} \right)^{2}}{\left( 1-cos\theta_{ti,max} \right)^{2}}$ | (19) |
| $\frac{d^{2}\theta_{ti}}{dt^{2}}=\left\{ \begin{matrix} \left( P_{ra}-P_{rv} \right)K_{p,ti}\cos\theta_{ti}-K_{f,ti}\frac{d\theta_{ti}}{dt}+K_{b,ti}Q_{ti}\cos\theta_{ti}-K_{v,ti}Q_{ti}\sin\left( 2\theta_{ti} \right), & Q_{ti}\geq0 \\ \left( P_{ra}-P_{rv} \right)K_{p,ti}\cos\theta_{ti}-K_{f,ti}\frac{d\theta_{ti}}{dt}+K_{b,ti}Q_{ti}\cos\theta_{ti}, & Q_{ti}<0 \end{matrix} \right.$ | (20) |

**S3.4. Right Ventricle**

The right ventricle was modeled using a time-varying elastance approach, capturing the interaction with the pulmonary circulation:

| $\frac{dV_{rv}}{dt}=Q_{ti}-Q_{po}$ | (21) |
| --- | --- |
| $P_{rv}=P_{rv,un}+E_{rv}\left( V_{rv}-V_{rv,un} \right)$ | (22) |
| $Q_{po}=\left\{ \begin{matrix} C_{Qpo}AR_{po}\sqrt{P_{rv}-P_{pas}}, & P_{rv}\geq P_{pas} \\ -C_{Qpo}AR_{po}\sqrt{P_{pas}-P_{rv}}, & P_{rv}<P_{pas} \end{matrix} \right.$ | (23) |
| $E_{rv}\left( t \right)=E_{rv,min}+\frac{E_{rv,max}-E_{rv,min}}{2}e_{v}\left( t \right)$ | (24) |
| $AR_{po}=\frac{\left( 1-cos\theta_{po} \right)^{2}}{\left( 1-cos\theta_{po,max} \right)^{2}}$ | (25) |
| $\frac{d^{2}\theta_{po}}{dt^{2}}=\left\{ \begin{matrix} \left( P_{rv}-P_{pas} \right)K_{p,po}\cos\theta_{po}-K_{f,po}\frac{d\theta_{po}}{dt}+K_{b,po}Q_{po}\cos\theta_{po}-K_{v,po}Q_{po}\sin\left( 2\theta_{po} \right), & Q_{po}\geq0 \\ \left( P_{rv}-P_{pas} \right)K_{p,po}\cos\theta_{po}-K_{f,po}\frac{d\theta_{po}}{dt}+K_{b,po}Q_{po}\cos\theta_{po}, & Q_{po}<0 \end{matrix} \right.$ | (26) |

**S4. Vascular System Modeling**

**S4.1 Systemic Circulation**

The systemic circulation was represented using combinations of resistance (R), compliance (C), and inertance (L) elements. These components accounted for viscous losses, vascular elasticity, and flow inertia, respectively:

| $\frac{dP_{sas}}{dt}=\frac{Q_{ao}-Q_{sas}}{C_{sas}}$ | (27) |
| --- | --- |
| $\frac{dQ_{sas}}{dt}=\frac{P_{sas}-P_{sat}-R_{sas}Q_{sas}}{L_{sas}}$ | (28) |
| $\frac{dP_{sat}}{dt}=\frac{Q_{sas}-Q_{sat}}{C_{sat}}$ | (29) |
| $\frac{dQ_{sat}}{dt}=\frac{P_{sat}-P_{svn}-\left( R_{sat}+R_{sar}+R_{scp} \right)Q_{sat}}{L_{sat}}$ | (30) |
| $\frac{dP_{svn}}{dt}=\frac{Q_{sat}-Q_{svn}}{C_{svn}}$ | (31) |
| $Q_{svn}= \frac{P_{svn}- P_{ra}}{R_{svn}}$ | (32) |

**S4.2 Pulmonary Circulation**

The pulmonary circulation was modeled using a similar R–C–L network, with parameters adjusted to reflect the lower pressure and resistance characteristics of the pulmonary vasculature:

| $\frac{dP_{pas}}{dt}=\frac{Q_{po}-Q_{pas}}{C_{pas}}$ | (33) |
| --- | --- |
| $\frac{dQ_{pas}}{dt}=\frac{P_{pas}-P_{pat}-R_{pas}Q_{pas}}{L_{pas}}$ | (34) |
| $\frac{dP_{pat}}{dt}=\frac{Q_{pas}-Q_{pat}}{C_{pat}}$ | (35) |
| $\frac{dQ_{pat}}{dt}=\frac{P_{pat}-P_{pvn}-\left( R_{pat}+R_{par}+R_{pcp} \right)Q_{pat}}{L_{pat}}$ | (36) |
| $\frac{dP_{pvn}}{dt}=\frac{Q_{pat}-Q_{pvn}}{C_{pvn}}$ | (37) |
| $Q_{pvn}= \frac{P_{pvn}- P_{la}}{R_{pvn}}$ | (38) |

**S4.3 For Ripples**

For Aortic Valve Closure

| $Q_{ao} = 0$ | (39) |
| --- | --- |

The pressure equation of the systemic aortic sinus becomes

| $C_{sas}\frac{dP_{sas}}{dt} =Q_{ao}-Q_{sas}$ | (40) |
| --- | --- |
| $C_{sas}\frac{dP_{sas}}{dt} =-Q_{sas}$ | (41) |
| $\frac{dP_{sas}}{dt} = \frac{{-Q}_{sas}}{C_{sas}}$ | (42) |

The downstream flow equation is

| $L_{sas}\frac{dQ_{sas}}{dt} =P_{sas} -P_{sat}-R_{sas}Q_{sas}$*​* | (43) |
| --- | --- |

Differentiating both sides with respect to time gives

| $L_{sas}\frac{d^{2}Q_{sas}}{{dt}^{2}} = \frac{dP_{sas}}{dt} - \frac{dP_{sat}}{dt} -R_{sas} \frac{dQ_{sas}}{dt}$ | (44) |
| --- | --- |

Substituting

| $\frac{dP_{sas}}{dt} = \frac{{-Q}_{sas}}{C_{sas}}$ | (45) |
| --- | --- |

Yields

| $L_{sas}\frac{d^{2}Q_{sas}}{{dt}^{2}} =\frac{{-Q}_{sas}}{C_{sas}}- \frac{dP_{sat}}{dt} -R_{sas} \frac{dQ_{sas}}{dt}$ | (46) |
| --- | --- |

Rearranging

| $L_{sas}\frac{d^{2}Q_{sas}}{{dt}^{2}}+R_{sas} \frac{dQ_{sas}}{dt}+ \frac{1}{C_{sas}}Q_{sas}= - \frac{dP_{sat}}{dt}$ | (47) |
| --- | --- |

If $P_{sat}$ varies approximately linearly after valve closure, then

| $\frac{\text{d}P_{sat}}{\text{dt}}\approx a$ | (48) |
| --- | --- |

And the equation becomes

| $L_{sas}\frac{d^{2}Q_{sas}}{{dt}^{2}}+R_{sas} \frac{dQ_{sas}}{dt}+ \frac{1}{C_{sas}}Q_{sas}= -a$ | (49) |
| --- | --- |

This has the same mathematical form as a forced mass-spring-damper equation

| $m\ddot{x}+c\dot{x}+kx =F_{0}$ | (50) |
| --- | --- |

**S5. Initial Conditions**

The initial conditions and model parameters used in the simulations are summarized in Tables 2–7.

| **Variable** | **Value (**$\boldsymbol{t}\mathbf{=}\boldsymbol{0}$**)** |
| --- | --- |
| $V_{la,0}$ | 60 ml |
| $V_{lv,0}$ | 130 ml |
| $V_{ra,0}$ | 39 ml |
| $V_{rv,0}$ | 110 ml |
| $P_{sas,0}$ | 100 mmHg |
| $Q_{sas,0}$ | 0 ml/s |
| $P_{sat,0}$ | 100 mmHg |
| $Q_{sat,0}$ | 0 ml/s |
| $P_{svn,0}$ | 10 mmHg |
| $P_{pas,0}$ | 20 mmHg |
| $Q_{pas,0}$ | 0 ml/s |
| $P_{pat,0}$ | 20 mmHg |
| $Q_{pat,0}$ | 0 ml/s |
| $P_{pvn,0}$ | 10 mmHg |
| $\theta_{mi,0}=\frac{d\theta_{mi,0}}{dt}$ | 0 rad |
| $\theta_{ao,0}=\frac{d\theta_{ao,0}}{dt}$ | 0 rad |
| $\theta_{ti,0}=\frac{d\theta_{ti,0}}{dt}$ | 0 rad |
| $\theta_{po,0}=\frac{d\theta_{po,0}}{dt}$ | 0 rad |

**Table 2**: Initial Conditions

The parameter values listed in the following tables were selected from previously published cardiovascular modeling studies and physiological measurements reported in the literature [1, 2]. The values were maintained within acceptable ranges and were not arbitrarily tuned to reproduce a specific reference waveform.

**S6. Cardiac parameters**

| **Parameter** | **Value** |
| --- | --- |
| $CQ_{ao}$ | 350 ml/(s·mmHg${}^{0.5}$) |
| $CQ_{mi}$ | 400 ml/(s·mmHg${}^{0.5}$) |
| $E_{lv,max}$ | 2.5 mmHg/ml |
| $E_{lv,min}$ | 0.07 mmHg/ml |
| $P_{lv,un}$ | 1 mmHg |
| $V_{lv,un}$ | 5 ml |
| $E_{la,max}$ | 0.25 mmHg/ml |
| $E_{la,min}$ | 0.15 mmHg/ml |
| $P_{la,un}$ | 1 mmHg |
| $V_{la,un}$ | 4 ml |
| $CQ_{po}$ | 350 ml/(s·mmHg${}^{0.5}$) |
| $CQ_{ti}$ | 400 ml/(s·mmHg${}^{0.5}$) |
| $E_{rv,max}$ | 1.15 mmHg/ml |
| $E_{rv,min}$ | 0.07 mmHg/ml |
| $P_{rv,un}$ | 1 mmHg |
| $V_{rv,un}$ | 10 ml |
| $E_{ra,max}$ | 0.25 mmHg/ml |
| $E_{ra,min}$ | 0.15 mmHg/ml |
| $P_{ra,un}$ | 1 mmHg |
| $V_{ra,un}$ | 4 ml |

**Table 3**: Cardiac Parameters

**S7. Systemic Circulation Parameters**

| **Parameter** | **Value** |
| --- | --- |
| $C_{sas}$ | 0.08 ml/mmHg |
| $R_{sas}$ | 0.003 mmHg·s/ml |
| $L_{sas}$ | 0.000062 mmHg·s${}^{2}$/ml |
| $C_{sat}$ | 1.6 ml/mmHg |
| $R_{sat}$ | 0.05 mmHg·s/ml |
| $L_{sat}$ | 0.0017 mmHg·s${}^{2}$/ml |
| $R_{sar}$ | 0.5 mmHg·s/ml |
| $R_{scp}$ | 0.52 mmHg·s/ml |
| $R_{svn}$ | 0.075 mmHg·s/ml |
| $C_{svn}$ | 20.5 ml/mmHg |

**Table 4:** Systemic Circulation parameters

**S8. Pulmonary Circulation Parameters**

| **Parameter** | **Value** |
| --- | --- |
| $C_{pas}$ | 0.18 ml/mmHg |
| $R_{pas}$ | 0.002 mmHg·s/ml |
| $L_{pas}$ | 0.000052 mmHg·s${}^{2}$/ml |
| $C_{pat}$ | 3.8 ml/mmHg |
| $R_{pat}$ | 0.01 mmHg·s/ml |
| $L_{pat}$ | 0.0017 mmHg·s${}^{2}$/ml |
| $R_{par}$ | 0.05 mmHg·s/ml |
| $R_{pcp}$ | 0.07 mmHg·s/ml |
| $R_{pvn}$ | 0.006 mmHg·s/ml |
| $C_{pvn}$ | 20.5 ml/mmHg |

**Table 5:** Pulmonary Circulation Parameters

**S9. Valve Dynamics Parameters**

| **Parameter** | **Value** |
| --- | --- |
| $K_{p,mi},K_{p,ao},K_{p,ti},K_{p,po}$ | 5500 s${}^{-1}$·mmHg |
| $K_{f,mi},K_{f,ao},K_{f,ti},K_{f,po}$ | 50 s${}^{-1}$ |
| $K_{b,mi},K_{b,ao},K_{b,ti},K_{b,po}$ | 2 rad/(s·ml) |
| $K_{v,mi},K_{v,ao},K_{v,ti}$ | 3.5 rad/(s·ml) |
| $K_{v,ao},K_{v,po}$ | 7 rad/(s·ml) |
| $\theta_{\max}$ | $5/12\pi$ rad |

**Table 6:** Valve Dynamics Parameters

**S10. Timing Parameters**

| **Parameter** | **Value** |
| --- | --- |
| $T_{ac}$ | $0.875 RR$ s |
| $T_{me}$ | $0.3 RR$ s |
| $T_{ce}$ | $\frac{3}{2}T_{me}$ s |

**Table 7**: Timing Parameters

**S11. Reproducibility**

All equations, parameters, and initial conditions required to reproduce the simulations are provided in this supplementary material. The model can be implemented using standard numerical solvers for ordinary differential equations.

**Reference**

[1] T. Korakianitis, Y. Shi, “Numerical simulation of cardiovascular dynamics with healthy and diseased heart valves”, *J Biomech* **2006**, *39* (11), 1964, <https://doi.org/10.1016/j.jbiomech.2005.06.016>.

[2] S. Scarsoglio, A. Guala, C. Camporeale, L. Ridolfi, “Impact of atrial fibrillation on the cardiovascular system through a lumped-parameter approach”, *Med Biol Eng Comput* **2014**, *52* (11), 905, <https://doi.org/10.1007/s11517-014-1192-4>.
